# Socioeconomic differentials in multimorbidity prevalence, health and social care expenditure among persons 65 years and older in region Stockholm, Sweden: a population-based study

**DOI:** 10.1136/bmjopen-2025-106179

**Published:** 2026-08-14

**Authors:** Megan Doheny, Bo Burström, Janne Agerholm, Ann Liljas

**Affiliations:** 1 Aging Research Center, Karolinska Institutet, Stockholm, Stockholm County, Sweden; 2 Global Public Health, Karolinska Institutet, Stockholm, Sweden; 3 Centre for Epidemiology and Community Medicine, Region Stockholm, Stockholm, Sweden

**Keywords:** Multimorbidity, Health Services, Health Services for the Aged, Health economics, SOCIAL MEDICINE

## Abstract

**Abstract:**

**Objectives:**

This study aims to assess socioeconomic differentials in the prevalence of multimorbidity among adults ≥65 years, health and social care expenditure and its effect on total care expenditure.

**Design:**

Population-based cross-sectional register study.

**Setting:**

Region Stockholm, Sweden.

**Participants:**

Persons ≥65 years (N=371 583), in Region Stockholm in 2019.

**Primary outcome:**

Total health and social care expenditure per individual in 2019.

**Secondary outcome:**

Prevalence and sociodemographic patterns of multimorbidity, differences in care expenditure across socioeconomic groups and the composition of services used.

**Results:**

Multimorbidity prevalence was 62.6% among adults aged ≥65 years (N=371 583), increasing with age and occurring earlier in lower income groups. Average total care expenditure increased from 34 346 Swedish Kronor (SEK) among individuals with no chronic conditions and 55 606 SEK among those with one condition to 143 063 SEK among those with ≥4 chronic conditions. Social care expenditure exceeded healthcare expenditure from age 80 years onwards. In adjusted gamma regression models, multimorbidity was associated with 11.6% higher total care expenditure (cost ratio (CR) 1.116, CI 1.114 to 1.118). Compared with the highest income group, expenditure was also higher among those in the lowest (CR: 1.038, CI 1.036 to 1.040) and second-lowest income groups (CR: 1.011, CI 1.009 to 1.013).

**Conclusions:**

Multimorbidity is increasingly prevalent in older ages and is associated with higher health and social care expenditure. This poses a challenge to organising and financing healthcare, and critically, social care services, in the future. More resources should be allocated to health promotion and prevention to delay onset, targeting lower socioeconomic groups. Alternative approaches to organising care that balance quality, equity and cost-effectiveness should be explored.

**STRENGTHS AND LIMITATIONS OF THIS STUDY**

- Population-based register data including all adults aged ≥65 years in Region Stockholm (N=371 583) provided high statistical power and reduced selection bias.
- Linkage of healthcare and social care registers enabled assessment of total care expenditure across care sectors.
- The cross-sectional study design limits causal inference and assessment of temporal changes in multimorbidity and care expenditure.
- Expenditure estimates did not include prescription drug costs or unpaid informal care.
- The cross-sectional study design limits causal inference and assessment of temporal changes in multimorbidity and care expenditure.

## Introduction

 As populations age, multimorbidity (ie, the coexistence of two or more chronic conditions) has become increasingly prevalent.^1 2^ According to global estimates, multimorbidity affects over one-third of adults and increases markedly in older age, with the highest rates observed in the oldest age groups.^3^ Multimorbidity is associated with elevated healthcare utilisation, functional decline, reduced quality of life and extensive care needs,^4 5^ placing a significant financial strain on health and social care systems.^6 7^

The distribution of multimorbidity is socioeconomically patterned, as individuals in disadvantaged groups tend to develop multimorbidity earlier and experience more severe disease trajectories and functional limitations.^5 6 8^ These limitations are likely to translate into premature and disproportionate use of health and social care services, thereby amplifying spending on care services.^9–11^ Nevertheless, research on multimorbidity-related care use and spending across socioeconomic groups remains limited. Such insights are essential for care planning and financing of health and social care systems in the context of population ageing, especially in welfare states that aspire to equitable access and care provision.

The Swedish health and social care system is universal and publicly funded through taxes, and while citizens pay a small fraction out-of-pocket, the maximum is capped by cost ceilings.^12^ Moreover, the provision of care is guided by *equal access for equal need,* regardless of age, sex or economic resources.^13 14^ The organisation of care is divided between national, regional and municipal levels. The national government sets directives and laws, the 21 regions are responsible for financing and delivering health and medical care, whereas the 290 municipalities are responsible for financing and delivering social care for older adults, including home help services and managing care homes. Regions and municipalities are autonomous, as they are governed by different laws and have separate information systems and financial budgets.^12^

The division of responsibilities and financial autonomy between regions and municipalities has resulted in a structurally fragmented care system in Sweden. This limits care coordination, despite multimorbidity individuals often requiring various combinations of both health and social care services.^10 11 15^ This fragmentation is further compounded by socioeconomic differences, as lower socioeconomic groups generally experience higher levels of multimorbidity and a greater reliance on multiple forms of care, while simultaneously encountering barriers associated with poor coordination.^16^ Together, these factors may help explain why few studies have examined the interaction between health and social care from a spending perspective in other European countries.^11 17 18^

This knowledge gap is becoming increasingly problematic given population ageing and the rising multimorbidity prevalence, which will place further pressure on already strained regional and municipal budgets.^7 9 16^ This study aims to: (a) estimate the prevalence of multimorbidity among adults 65 years and older in Region Stockholm across age, sex and socioeconomic groups; (b) describe expenditure on health and social care services related to multimorbidity and (c) examine the effect of multimorbidity on total care expenditure.

## Methods

A population-based study was performed using individually linked register data via encrypted serial numbers. The total population register *Registret över totalbefolkning* (RTB)^19^ was used to define the study population, which included (N=3 71 583) adults aged ≥65 years registered as living in Region Stockholm on 1 January 2019. Annual health and social care expenditure associated with multimorbidity was measured in 2019 to limit the impact of the COVID-19 pandemic. Moreover, cumulative care spending was not considered because disease duration varies substantially between individuals. The study population was linked to the Cause of Death Register to identify those who died during 2019 (n=13 218), based on the registered date of death, and care expenditures were measured for the 12 months prior to the date of death.

Demographic factors were obtained from RTB. Sex was categorised into male or female, age was measured using year of birth, and country of birth was dichotomised into Sweden or other. This was linked to the Longitudinal Integrated Database for Social Insurance and Labour Market Studies (LISA), a collection of variables from several different population-based registers, which are individually linked and used to measure socioeconomic position (SEP).^20^ Income was measured using net annual equivalised household income in 2018 and was ranked into quintiles from lowest to highest, based on the distribution of income among all inhabitants ≥18 years in Region Stockholm. Income was selected as the main socioeconomic indicator as it has been demonstrated to be strongly associated with late-life health compared with other indicators.^21^ However, the primary income source in this population is pensions, which, while reflecting prior labour market participation, may not fully capture SEP in older ages.^22^ As such, education level was also assessed (measured in LISA) and categorised as primary (<9 years), secondary (9–12 years) and postsecondary (>12 years and used to conduct a sensitivity analysis (online supplemental table S2).

### Multimorbidity

Multimorbidity was measured using the classification framework developed by Calderon-Larranaga *et al*.^23^ This framework is based on a comprehensive list of chronic conditions identified using ICD-10 codes. Diagnoses were considered chronic if they had a prolonged duration, resulted in a residual disability or reduced quality of life, or required a long period of care, treatment or rehabilitation. A total of 918 diagnoses fulfilled these criteria and were grouped into 60 chronic disease categories according to predefined clinical criteria.^23^ The data used to measure multimorbidity were obtained from the Region Stockholm Healthcare administrative database (“Vårdanalys”VAL by Swedish acronym). VAL is composed of several different registers and includes information on all inpatient, outpatient, primary care, home healthcare and publicly financed private healthcare services reimbursed by Region Stockholm. Multimorbidity was measured using ICD-10 codes recorded in outpatient and inpatient care between 2016 and 2018. Because multimorbidity reflects a long-standing and cumulative disease burden, chronic conditions diagnosed prior to 2016 could still be captured through healthcare contacts during the observation period. The number of chronic conditions per individual was categorised as none, one, two, three or four or more. To assess the socioeconomic and demographic differences in multimorbidity and care expenditure, multimorbidity was defined as the presence of two or more chronic conditions, without distinguishing between specific disease combinations or patterns.

### Health and social care expenditure

Healthcare expenditure was measured using data obtained from region inpatient and outpatient care as well as private practice registers within VAL. Expenditure on outpatient care (ie, primary care, rehabilitation, occupational therapy), emergency care, outpatient specialist care (ie, consultations and treatments delivered by physicians in outpatient clinics) and home healthcare services was calculated by multiplying the registered cost weight for each care contact, representing a standardised reimbursement rate to the care provider, by its corresponding fixed monetary value in 2019.^24^ Expenditure on publicly funded privately provided outpatient specialist care was measured using the same approach. Home healthcare expenditure was estimated according to the type (basic or advanced) and the duration. Cost weight data were missing for 4% of outpatient care contacts. In these instances, expenditure was estimated using the modal cost weight for each contact, derived by care type, contact form and provider type (physician vs other healthcare professional). Inpatient care expenditure was calculated using diagnosis-related group (DRG) weights, updated annually by the National Board of Health and Welfare. DRG weights were assigned to all planned and unplanned inpatient care contacts (somatic, surgical, geriatric and psychiatric) and multiplied by the monetary value per DRG unit, adjusted to 2019 price levels.

Social care expenditure was estimated using data retrieved from the Swedish Social Service Register (SOL), which collects monthly data on publicly funded social care services, including home-help (domestic and/or personal care) and care home placement.^25^ Expenditure on home-help was estimated by multiplying the number of hours of home-help granted per month, as recorded in SOL, by the municipal price per hour. Care home expenditure was estimated by multiplying the number of months an individual was registered as residing in a care home in 2019, as recorded in SOL, by the average monthly cost per care home resident to municipalities, derived from data provided by the Swedish Association of Local Authorities and Regions.^26^

### Patients and public involvement

Patients or the public in general were not involved in this research.

### Analysis

Multimorbidity prevalence was estimated in the study population, stratified by sex, country of birth and income groups and plotted across age in years to visually assess variation. Mean care expenditure was calculated across age groups, and the composition of care services was compared between individuals with multimorbidity and those without (0 or 1 chronic conditions). All expenditures are presented at 2019 price levels (9.46 SEK=1 USD).

Generalised linear models (GLM) with a gamma distribution and log link were used to examine the association between total care expenditure and multimorbidity, adjusting for socioeconomic and demographic factors.^27^ The outcome was highly skewed, and model fit was assessed by comparing the log-likelihood and Akaike information criteria as well as plots of predicted values and residuals, the outcome was transformed to the natural logarithmic scale (online supplemental figure S1) (online supplemental table S1). Regression coefficients were exponentiated and are interpreted as the relative change (cost ratios (CR)) in total care expenditure associated with a one-unit change in continuous covariates, or relative to a reference group for categorical variables. Model 0 examined the unadjusted effect of multimorbidity on total care expenditure. Model 1 additionally adjusted for income group. Model 2 further adjusted for age, sex and country of birth to account for potential confounding demographic factors. All analyses were performed in STATA V.15.1.

## Results

Table 1 describes the (N=3 71 583) adults aged ≥65 years registered as residing in Region Stockholm in 2019, 54.8% were female, the mean age was 74.8 (±7.4) years and 62.6% were multimorbid (≥2 chronic conditions). Multimorbidity was higher among females 65–84 years (61.3% vs 58.8%) but was exceeded by males ≥85 years (79% vs 82.2%) (online supplemental figure S2). Multimorbidity was more prevalent among lower income groups at 65–79 years (60% vs 49.5%) compared with the highest income groups until 80 years (75.9% vs 79.5%) (online supplemental figure S3file). In both sexes, multimorbidity was more prevalent and occurred at younger ages in lower income groups than in the highest income group (online supplemental figure S4).

**Table 1:** Socioeconomic and demographic characteristics of adults aged≥65 years living in Region Stockholm in 2019, and average care expenditures presented by healthcare, social care and total care expenditure

	Total population	Average care expenditure in Swedish Krona (SEK)		
	N=371 583	Healthcare	Social care	Total care
	(%)	Mean C (SE)	Mean C (SE)	Mean C (SE)
Average expenditure per person		41 581 (178)	41 516 (284)	83 098 (353)
Sex				
Male	45.2	42 857 (289)	29 277 (353)	72 134 (481)
Female	54.8	40 530 (224)	51 601 (427)	92 131 (506)
Age groups				
65–69 years	27.6	29 137 (348)	7545 (225)	36 682 (435)
70–74 years	28.4	35 993 (328)	14 120 (303)	50 113 (475)
75–79 years	19.8	44 635 (393)	27 581 (502)	72 216 (681)
80–84 years	12.1	54 768 (511)	61 955 (960)	116 723 (1144)
85–89 years	7.3	64 704 (664)	128 624 (1724)	193 329 (1914)
≥90 years	4.9	64 700 (742)	267 389 (2903)	332 089 (3006)
Income groups				
Group 1 (lowest)	23.6	49 439 (399)	78 362 (782)	127 802 (919)
Group 2	26.8	48 036 (370)	49 689 (591)	97 726 (734)
Group 3	16.4	38 822 (428)	24 910 (541)	63 732 (727)
Group 4	14.5	33 839 (436)	15 356 (458)	49 195 (660)
Group 5 (highest)	18.2	31 001 (352)	18 002 (469)	49 003 (609)
Missing	0.4	19 841 (1624)	18 809 (3034)	38 650 (3664)
Country of birth				
Other	21.9	39 787 (373)	40 110 (581)	79 897 (734)
Sweden	78.1	42 084 (203)	41 911 (325)	83 995 (402)
Multimorbidity				
None	19.5	14 539 (226)	19 807 (492)	34 346 (556)
1 condition	17.9	24 760 (310)	30 846 (636)	55 606 (722)
2 conditions	17.2	32 760 (363)	30 164 (609)	62 923 (731)
3 conditions	14.5	41 437 (428)	37 333 (710)	78 771 (860)
≥4 conditions	30.9	73 377 (430)	69 686 (612)	143 063 (792)

The exchange rate at average price level in 2019 (9.46 SEK = 1 US$). Source: The Central Bank of Sweden https://www.riksbank.se/en-gb

(%), proportion; Mean C, average expenditure.

**Figure 1:**
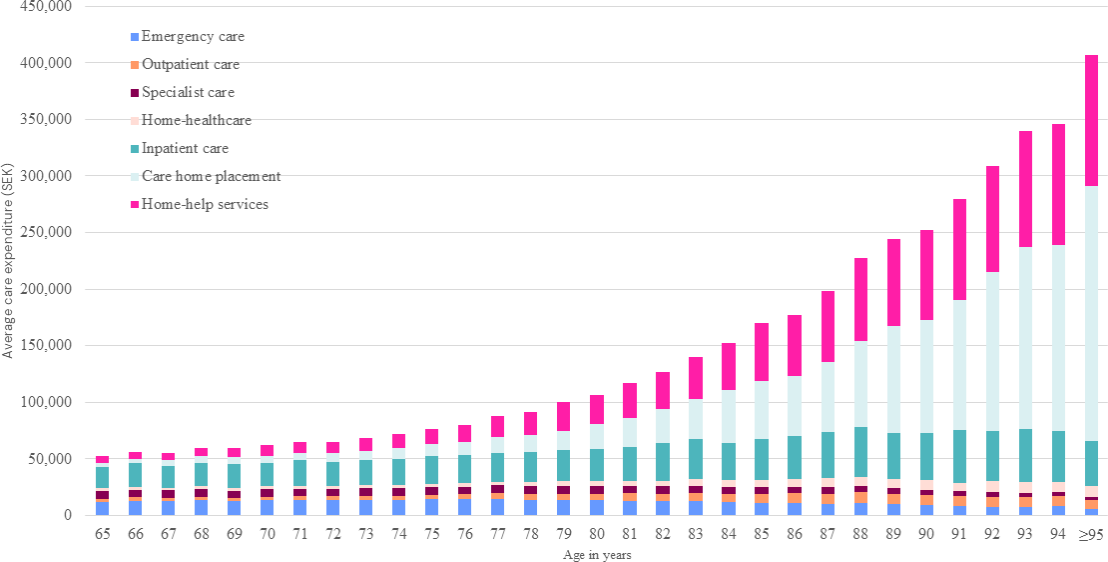
Mean expenditure on health and social care services by age among adults≥65 years in Region Stockholm in 2019.

Average healthcare expenditure was 41 581 SEK and social care was 41 516 SEK per person. Total care expenditure increased with age, from 36 682 SEK at 65–69 years to 332 089 SEK ≥90 years, and the composition of health and social care services used varied across age groups (see figure 1). Social care expenditure exceeded healthcare expenditure from age 80 years onwards (61 955 vs 54 768 SEK at ages 80–84 years) and accounted for most expenditure among those aged ≥90 years (267 389 vs 64 700 SEK). Males incurred higher average expenditure on healthcare (42 857 vs 40 530 SEK), while females incurred higher social care expenditure (29 277 vs 51 601 SEK). Lower income groups incurred higher total care expenditures compared with the highest (127 802 vs 49 003 SEK), driven primarily by social care expenditure, see online supplemental figure S5. There were n=29 716 individuals who incurred zero care expenditure, reflecting non-use of publicly funded health and social care services rather than missing data (see online supplemental table S2).

**Figure 2:**
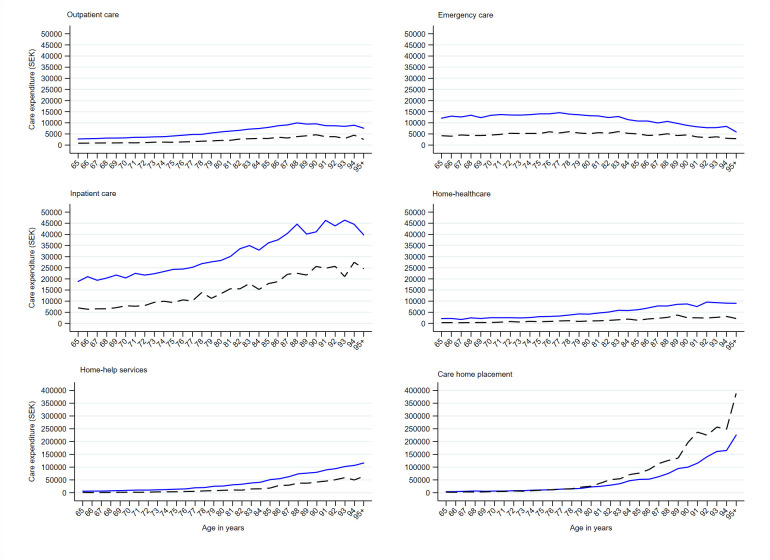
Average care expenditure for different health and social care services by multimorbidity status and age in adults≥65 years in Region Stockholm in 2019. Solid blue line=multimorbidity (≥2 chronic conditions), black dashed line=no multimorbidity (0–1 chronic condition).

Total care expenditure increased with the number of chronic conditions, from 34 346 SEK among those with no chronic conditions to 143 063 SEK among those with ≥4 conditions. Individuals with multimorbidity incurred higher average expenditure on inpatient, outpatient, home healthcare and home-help expenditure compared with those without multimorbidity across ages, see figure 2. Care home expenditure was higher among non-multimorbid individuals aged ≥80 years (106 974 vs 66 214 SEK), largely reflecting dementia-related care home placements, see online supplemental figure S6.

The GLM regression coefficients were exponentiated and interpreted as the relative change in total care expenditure (table 2). In model 0, multimorbidity was associated with a 14.7% higher total care expenditure (CR:1.147, CI 1.145 to 1.149). In model 1, significant differences between income groups could be observed, those in income group 1 (lowest) incurred 7.5% and income group 2 incurred 4.7% higher total care expenditure compared with those in income group 5 (highest). In model 2, adjusting for both socioeconomic and demographic factors, multimorbidity remained significantly associated with 11.6% higher average total care expenditure (CR:1.116, CI 1.114 to 1.118). Compared with the highest income group, expenditure was higher in income group 1 (CR: 1.038, CI 1.036 to 1.040) and income group 2 (CR:1.011, CI 1.009 to 1.013). Age was associated with 0.7% higher total care expenditure. Country of birth was associated with only slight differences in total care expenditure, with those born outside of Sweden incurring 1% lower total care expenditure (CR: 0.990, CI 0.968 to 0.992) compared with those born in Sweden. In the sensitivity analysis, the direction of association between education level and total care expenditure was consistent with the analysis when using income, although effect sizes differed (online supplemental table S2).

**Table 2:** The exponentiated regression coefficients assessing the association between total care expenditure and multimorbidity

	Model 0		Model 1		Model 2	
	Relative cost ratio	95% CI	Relative cost ratio	95% CI	Relative cost ratio	95% CI
Multimorbidity						
0–1 chronic conditions	ref		ref		ref	
≥2 chronic conditions	1.147	(1.145 to 1.149)	1.140	(1.138 to 1.141)	1.116	(1.114 to 1.118)
Income group						
Group 5 (highest)			ref		ref	
Group 1 (lowest)			1.075	(1.073 to 1.077)	1.038	(1.036 to 1.040)
Group 2			1.047	(1.046 to 1.050)	1.011	(1.009 to 1.013)
Group 3			1.016	(1.014 to 1.019)	1.001	(0.998 to 1.002)
Group 4			1.000	(0.998 to 1.003)	0.998	(0.996 to 1.001)
Age in years					1.008	(1.007 to 1.008)
Sex						
Male					ref	
Female					1.001	(0.999 to 1.002)
Country of birth						
Sweden					ref	
Other					0.990	(0.968 to 0.992)

The exponentiated regression coefficients from a generalised linear model with a gamma distribution and a log link function. The regression models were performed on n=341 123 observations.

Model 0, multimorbidity; Model 1, model 0 + income group; Model 2, model 1 + age in years + sex + country of birth.

## Discussion

This study examined the prevalence of multimorbidity among adults aged 65 years and older in Region Stockholm in 2019, described expenditure on health and social services in relation to multimorbidity, and estimated the effect of multimorbidity on total care expenditure. In the study population, 62.6% met the criteria for multimorbidity. Prevalence increased with age and was higher among females, while lower income groups experienced an earlier onset of multimorbidity. There was a notable shift, whereby expenditure on social care services began to exceed expenditure on healthcare services from age 80 years onwards. Total care expenditure increased with the number of chronic conditions, ranging from an average of 55 606 SEK among those with one chronic condition to 143 063 SEK among those with four or more. Multimorbid older adults incurred comparably higher expenditure on inpatient care, outpatient care, emergency care, home healthcare and home-help services. The multivariate analysis showed that those with multimorbidity incurred on average 12% higher total care expenditure compared with those with zero or one chronic condition after adjusting for socioeconomic and demographic factors.

This study observed a higher prevalence rate (62.6%) of multimorbidity compared with what was estimated previously.^3^ This can be attributed to differences in the age range of the study population, the number of chronic conditions included in the definition of multimorbidity, tests administered to determine the presence or absence of disease and data variability.^2 7 23^ Nonetheless, multimorbidity is increasingly prevalent in older ages^28^ and it is necessary to consider whether increased life expectancy will be spent with good or ill health.^9 11 16 17^ A German study observed that the prevalence of multimorbidity was continuously rising between 2010 and 2015, and that life expectancy gains were accompanied by more years in poor health.^29^ Multimorbidity was more prevalent among females at earlier ages compared with males. Moreover, there were sex differences in service-specific expenditure, where females incurred higher social care expenditure, whereas males incurred slightly higher healthcare expenditure. These differences in expenditure might be attributed to marked differences in disease progression, care-seeking behaviours, availability of informal support and life expectancy as well as broader structural and social factors within care systems.^1 16 17 30^

Lower income groups experienced an earlier onset of multimorbidity compared with higher income groups, consistent with the previous research.^5 6 8 10^ These socioeconomic differences likely reflect accumulated disadvantages, including lifestyle factors and living and working conditions that contribute to earlier development of chronic disease.^6 31 32^ Strengthening prevention and health promotion efforts targeted at lower socioeconomic groups may, therefore, yield substantial benefits, given that individuals with fewer chronic conditions had markedly lower health and social care expenditure. Higher income groups, by contrast, experienced a later onset of multimorbidity and lower average expenditure across age ranges, a pattern consistent with the previous studies, which suggested that higher socioeconomic groups remain healthy for longer.^32 33^ This pattern could be interpreted as a compression of morbidity into a shorter period towards the end of life, during which care tends to be intensive and costly. This interpretation is supported by previous Swedish studies, which observed that higher SEP is associated with higher healthcare expenditure in the last year of life.^34 35^ The socioeconomic variation observed in this study may arise from inequities in access to care, differences in diagnostic practices or the availability of informal care resources. Sex and socioeconomic differences in expenditure may also reflect broader social and structural factors within the health and social care systems.^10 16 36^

The pattern of care expenditure varied across age, with social care exceeding healthcare expenditure in the oldest age groups towards the end of life. Despite social care being an important contributor to total care expenditure, it has not been extensively studied in relation to multimorbidity.^11 17 18^ This gap likely reflects variation in welfare state structures across countries, which differs in how responsibility for financing and delivering social care is assigned, constraining data comparability.^9 11 16^ Swedish social care services are predominantly tax-funded at the municipal level, with regulated user fees for home-help and care home placements.^12^ However, ensuring adequate capacity to meet the needs of an ageing population remains a challenge, given workforce shortages and reductions in care home placements.^37^ Consistently, individuals with multimorbidity incurred higher expenditure on healthcare and home-help services, whereas those with zero or one chronic condition accounted for a disproportionately higher share of care home expenditure, this increase is partly due to placements attributed to cognitive impairment and dementia.^38^ A previous study identified distinct end-of-life care expenditure patterns that differed primarily by social care use, with the largest group characterised by higher spending on home-help and healthcare services, while the second largest group had high care home expenditure and comparatively lower healthcare costs.^39^ Together, these findings demonstrate the potential for social care interventions to influence healthcare expenditure and highlight the need to review the planning and organisation of health and social care services to ensure the most effective use of resources in Sweden.

### Strengths and limitations

This study used high-quality population-based registers that included all inhabitants aged 65 and over in Stockholm County and enabled the measurement of multimorbidity, health and social care expenditure, and socioeconomic and demographic factors.^19 20 25 40^ However, care expenditure was measured during a single calendar year and therefore this study provides a snapshot of the health and social care expenditures associated with multimorbidity. As care needs and treatment intensity across disease trajectories vary, these estimates do not capture cumulative costs or changes in expenditure associated with disease progression.^9 41^ Furthermore, there were no data available to estimate prescription drug expenditure, which is a key cost driver of multimorbidity.^42^

Multimorbidity was measured using healthcare utilisation data in the years prior, this limits our ability to assess incidence of multimorbidity.^9^ Multimorbidity was defined according to the number of chronic conditions.^2 23^ Consequently, the analysis did not account for specific disease combinations, interactions between conditions or differences in disease severity, which may influence health and social care expenditure. A systematic review found that some multimorbidity combinations are associated with disproportionately high costs, driven by frequent hospitalisations and extensive care needs, including respiratory and mental health conditions, diabetes with cardiovascular disease and cancer with mental health conditions.^17^ Future research should explore disease combinations linked to higher health and social care expenditure, to identify high-need patients and inform targeted, cost-effective service planning.

The findings’ generalisability requires additional consideration, as care organisation is decentralised in Sweden, where regions and municipalities are highly autonomous in the financing and organising of health and social care services to their local populations.^15^ Additionally, rural–urban differences could not be examined meaningfully in this study, which is important as there are differences in care resources available.^43^ However, the strength of focusing on Region Stockholm is that expenditure for primary care and home healthcare can be estimated, which is not available nationally.^40^ Our findings are relevant in Sweden and in other countries with ageing populations, as they demonstrate the care resources used in relation to the prevalence of multimorbidity.

In conclusion, multimorbidity is highly prevalent among older adults and is associated with substantially higher health and social care expenditure, with social care costs exceeding healthcare costs towards the end of life. Rapidly ageing populations place growing pressure on the planning, organisation and financing of both healthcare and social care systems, with significant implications for future resource allocation. Our findings suggest the need to allocate more resources to health promotion and disease prevention, focusing on lower socioeconomic groups, may delay the onset of multimorbidity and reduce inequities in health and care expenditure. Further research is needed to explore alternative approaches to integrating health and social care resources in ways that balance care quality, equity and cost-effectiveness to meet the complex needs of ageing populations.

## Supplementary material

10.1136/bmjopen-2025-106179 online supplemental file 1

## Data Availability

Data may be obtained from a third party and are not publicly available.
