# Supplementary material for: Socioeconomic differentials in multimorbidity prevalence, health and social care expenditure among persons 65 years and older in region Stockholm, Sweden: a population-based study
Source: BMJ Open. 2026 Aug 14;16(8):e106179. doi: 10.1136/bmjopen-2025-106179 (PMC13479488; doi:10.1136/bmjopen-2025-106179)
Supplement: Supplementary file 1 [file bmjopen-2025-106179.reviewer_comments.pdf]

PEER REVIEW HISTORY

BMJ Open publishes all reviews undertaken for accepted manuscripts. Reviewers are asked to complete a checklist review form and are provided with free text boxes to elaborate on their assessment. These free text comments are reproduced below.

ARTICLE DETAILS

Title (Provisional)

Socioeconomic differentials in multimorbidity prevalence, health and social care expenditure among persons 65 years and older in Region Stockholm, Sweden: A population-based study

Authors

Doheny, Megan Marie; Burström, Bo; Agerholm, J; Liljas, Ann

VERSION 1 - REVIEW

**Reviewer**	**1**
**Name**	**Hamashima, Yuri**
**Affiliation**	**University of Bristol, NIHR ARC West, Population Health Sciences**
**Date**	**19-Jan-2026**
**COI**	**None**

Thank you very much for the opportunity to review this paper. Although this study addresses an important topic and uses a high-quality, large registry with considerable potential for future research, the overall quality of the current manuscript does not yet meet the standards expected by BMJ Open. I outline several key suggestions below that may perhaps be helpful for revision.

First, the manuscript would benefit from clearer definition and justification of the socioeconomic indicators used in the analysis. Given that socioeconomic differences are placed at central to the study, I recommend that the authors briefly discuss the conceptual challenges of using income as a socioeconomic indicator for older populations, potentially in the discussion section.

Related to this point, the authors may wish to comment on the potential relationship between income and multimorbidity status. For example, greater disease burden may affect income among older individuals who remain in paid employment. In addition, it would be useful to clarify whether the analysis considered the potential role of social care interventions in mitigating increases in healthcare expenditure (for instance, whether receipt

of home care might enable earlier identification of health deterioration and thereby potentially reduce downstream healthcare costs).

The background section could be more concise and focused. While the authors mentioned a lack of understanding regarding the relationship between multimorbidity and long-term expenditure set in Sweden, the research gap could be articulated more clearly. It would be helpful to indicate whether there are any current or recent policy initiatives or reforms in Sweden that make this research timely. As the study is situated in the Swedish context, the background section would also be strengthened by providing more detailed information on Swedish healthcare and long-term care financing and funding arrangements, rather than briefly mentioning this in the discussion section.

The estimation of private and social care costs is an interesting aspect of the study. It might be helpful to report how many older individuals across the different income groups use private services and to discuss whether and how such use contributes to differences in the long-term expenditures across the income groups. In addition, providing a list of the types of services included under health and social care expenditure would aid the interpretation of the results.

The authors should also clarify the rationale for selecting 2019 and focusing on a single year. If this choice was influenced by substantial policy changes around the period, this needs to be stated. While the authors acknowledge the limitation of focusing on a single region, the interpretation of findings might be enhanced by considering rural-urban differences among older individuals (if feasible).

**Reviewer**	**2**
**Name**	**Wang, Yueqing**
**Affiliation**	**Peking Union Medical College, Department of Chronic Disease**
**Date**	**14-Feb-2026**
**COI**	**None**

Thank you for the opportunity to review this insightful paper. The authors' analysis of the composition of total care expenditure, including sex and socioeconomic differences, is undoubtedly a critical aspect of multimorbidity, which remains a pressing public health challenge.

I have a few comments towards this research work.

Major comments:

1. Multimorbidity is defined as the cumulative diagnoses between 2016 and 2018, while care expenditure is limited to 2019, as stated in the abstract. Why was the cumulative

expenditure from 2016 not considered? How can the expenditure from a single year accurately capture the overall burden, given that treatment costs vary significantly with disease progression?

2. The measurement of multimorbidity should go beyond simply counting the number of diseases and consider the types of diseases and their interactions. For instance, a random combination like skin rash and hypertension differs significantly from a clinically relevant combination such as hypertension and coronary disease, which has greater impacts on health outcomes and costs. The lack of analysis on specific combinations limits the study's conclusions. The sharp rise in care expenditure for patients with 4+ comorbidities may be driven by cardiovascular, metabolic, or respiratory conditions, particularly in older populations. Identifying and analyzing the most common multimorbidity combinations, their association with care expenditure, and comparing these to average costs would provide deeper insights and support targeted prevention and management strategies.

3. "males have higher healthcare expenditures while females incur higher social care expenditures " and "the relative changes of care expenditure over time across different socio-economic groups" suggest that these patterns may be influenced by broader social and economic dynamics (healthcare access inequities, diagnostic bias or societal stereotypes, etc.) . These results should be thoroughly discussed to uncover the underlying drivers, assess their implications for policy and practice in healthcare and social care systems.

4. While the research highlights dementia as a major driver of care home placement expenditure, this conclusion may be biased due to the lack of investigation into the disease types within multimorbidity. Dementia is undoubtedly a critical condition contributing to higher expenditures, other conditions or combinations of diseases may also significantly drive care home placement costs. Additionally, it would be valuable to explore multimorbidity patterns within dementia patients compared to those without to better understand the interplay between dementia and other comorbidities. Why was dementia singled out in this analysis?

Minor commentes:

1. "specialist outpatient care" is not clearly defined or not with listed examples;

2. I would recommend the authors thoroughly review the manuscript for typos, grammatical errors, and inconsistencies in symbols or formatting. For example, in Introduction line 12, "with multimorbidity(11).." has duplicated comma.

3. In the Introduction, the burden of multimorbidity is repeatedly described as being associated with higher healthcare utilization and negative impacts on health and social care. It would be beneficial to present this information more concisely to avoid redundancy and enhance the clarity of the narrative.

4. Any historical diagnoses before 2016 could be possible.How to deal with this scenario?

5. Dose the Region Stockholm Healthcare administrative database (VAL) fully capturesall inpatient care, outpatient care, and private practice records, or only partially. If the database does not include all records, this limitation should be explicitly stated. Regarding the 297,116 patients without expenditure data, please clarify whether this is due to data issues, such as incomplete linkage or missing records, or other factors. When linking multiple databases, it is critical to perform rigorous data cleaning and quality assurance to ensure the integrity and reliability of the data used for analysis. Explicitly documenting these steps will help ensure high-quality data and robust conclusions.

6. "Model 0" is not defined in Method. The 2nd and 3rd phragraph of Results have mainly state as "higher" or "lower", but no explict numbers ever listed.

VERSION 1 - AUTHOR RESPONSE

Reviewer 1, comment: First, the manuscript would benefit from clearer definition and justification of the socioeconomic indicators used in the analysis. Given that socioeconomic differences are placed at central to the study, I recommend that the authors briefly discuss the conceptual challenges of using income as a socioeconomic indicator for older populations, potentially in the discussion section. Related to this point, the authors may wish to comment on the potential relationship between income and multimorbidity status. For example, greater disease burden may affect income among older individuals who remain in paid employment.

Response: Thank you for this valuable suggestion. Socioeconomic position (SEP) in this study was operationalized using equivalised household income, which captures material resources and is commonly used in register-based studies among older populations. In this age group income is primarily derived from pensions, which in turn are shaped by earlier labour market participation, occupation and educational attainment, and thus, can still reflect long-term socioeconomic advantage and disadvantage.

At the same time, we acknowledge important conceptual challenges when using income as an SEP indicator in older populations, particularly, after retirement, when income may no longer directly reflect current labour market position. This limitation was not well discussed in the manuscript, particularly, the potential reverse causation, whereby a higher disease burden may influence income among older adults who remain in paid employment.

To assess the robustness of our findings, we conducted a sensitivity analysis using the education level (highest educational attainment measured in the LISA register) as an alternative SEP indicator. The direction of association with total care expenditure was consistent with the main analysis using income, although effect sizes differed. This suggests that the observed socioeconomic gradients are not just driven by the choice of measure of SEP but might reflect broader inequalities in health and social care needs.

Action: Justification for the use of income as SEP indicator has been added to the Methods section and references have been added to support this decision.

Income was selected as the main socioeconomic indicator as it has been demonstrated to be strongly associated with late-life health compared to other indicators (Darin-Mattsson et al, 2017). However, as the primary income source in this population is pensions, which while reflecting prior labour market participation, may not fully capture SEP in older ages (REF). As such, education level was measured in LISA and categorised as primary (< 9 years), secondary (9–12 years) and post-secondary (> 12 years and used to conduct a sensitivity analysis.

Action: Level of education was assessed, and a sensitivity analysis has been conducted. The results have been included as supplemental material, see Table S2.

Reviewer 1, comment: In addition, it would be useful to clarify whether the analysis considered the potential role of social care interventions in mitigating increases in healthcare expenditure (for instance, whether receipt of home care might enable earlier identification of health deterioration and thereby potentially reduce downstream healthcare costs).
Response: Thank you for this insightful comment. We agree that social care interventions may influence healthcare expenditures by supporting daily activities, addressing care needs and preventing deterioration. We have added a paragraph to the Discussion relating our findings to this issue. As in this study, individuals with extensive social care use i.e., those residing in care homes incurred higher social care and comparatively lower expenditure on inpatient and specialist outpatient expenditure at older ages, suggesting a potential substitution effect. This interpretation is consistent with our recent study, in which we identified distinct patterns of health and social care expenditure during the last year of life, which highlighted were individuals who incurred the highest expenditure for municipal care homes and lower healthcare expenditure. While those in the highest spending group incurred the highest expenditures for home-help services as well as healthcare services.
Action: The following text has been added to the discussion section and additional reference added.
Additionally, there were sex differences in service specific expenditure with females incurring higher expenditure on social care services whereas males incurred slightly higher healthcare expenditure. These differences in expenditure might be attributed to marked differences in disease progression, care-seeking behaviours, availability of informal support, and life expectancy.

Reviewer 1, comment: The background section could be more concise and focused. While the authors mentioned a lack of understanding regarding the relationship between multimorbidity and long-term expenditure set in Sweden, the research gap could be articulated more clearly.

Response: Thank you for this helpful suggestion, the Introduction has been revised to more clearly describe the prevalence of multimorbidity, its socioeconomic patterning, and the implications for health and social care utilization and expenditure. This revision strengthens the motivation for examining differences in multimorbidity and related care costs across socioeconomic and demographic groups.
Action: Text added to the introduction.
As populations age, multimorbidity (i.e., the coexistence of two or more chronic conditions) has become increasingly prevalent. According to global estimates multimorbidity affects over one-third of adults and increases markedly in older age, with the highest rates observed in the oldest age groups. Multimorbidity is associated with elevated healthcare utilization, functional decline, reduced quality of life, and extensive care needs, placing a significant financial strain on health and social care systems. The distribution of multimorbidity is socioeconomically patterned, as individuals in disadvantaged groups tend to develop multimorbidity earlier, and experience more severe disease trajectories and functional limitations. These limitations are likely to translate into premature and disproportionate use of health and social care services, thereby amplifying spending on care services. Nevertheless, research on multimorbidity related care use and spending differs across socioeconomic groups, particularly, across health and social care remains limited. Such insights are essential for care planning and financing of health and social care systems in the context of population ageing, especially in welfare states that aspire to equitable access and care provision.

Reviewer 1, comment: It would be helpful to indicate whether there are any current or recent policy initiatives or reforms in Sweden that make this research timely.
Response: We thank the reviewer for this suggestion. To better situate the study within the Swedish policy context, we have added text to the Background section outlining the core equitable principles that govern the Swedish health and social care system, in particular the principle of "equal access for equal need," regardless of age, sex, or economic resources. This principle is central to ongoing policy discussions concerning fairness, efficiency, and resource allocation within healthcare and social care, especially in the context of population ageing and increasing multimorbidity. As equity is one of the key principles guiding the provision of care these guiding principles, and this study is timely in terms of upholding the goal of the system in light of changing demographics and care needs..
Action: The following text has been added to the Background outlining the equitable principles guiding the care systems.
Moreover, the provision of care is guided by *"Equal access for equal need",* regardless of age, sex or economic resources.

Reviewer 1, comment: As the study is situated in the Swedish context, the background section would also be strengthened by providing more detailed information on Swedish healthcare and long-term care financing and funding arrangements, rather than briefly mentioning this in the discussion section.
Response: Thank you for this comment. To better contextualise this study, we have expanded the description of the Swedish health and social care system. The added text outlines the universal and tax-funded nature of the system, the role of cost ceilings for out-of-pocket payments, the equitable principles guiding the provision of care, and the division of responsibilities across national, regional, and municipal levels. We also clarify the organizational and financial separation between healthcare and social care, and highlight the relevance of this structure for studying care expenditure.
Action: Text added to the background section.
The Swedish health and social care system is universal and publicly funded through taxes, and while citizens pay a small fraction out-of-pocket, the maximum is capped by cost ceilings. Moreover, the provision of care is guided by *"equal access for equal need",* regardless of age, sex or economic resources. The organization of care is divided between national, regional and municipal levels. The national government sets directives and laws, the 21 regions are responsible for financing and delivering health and medical care, whereas the 290 municipalities are responsible for financing and delivering social care for older adults, including home-help services and the management of care homes. Regions and municipalities are autonomous, as they are governed by different laws and have separate information systems and financial budgets.

Reviewer 1, comment: The estimation of private and social care costs is an interesting aspect of the study. It might be helpful to report how many older individuals across the different income groups use private services and to discuss whether and how such use contributes to differences in the long-term expenditures across the income groups.
Response: This study focused on estimating public expenditure on healthcare services and therefore included only private specialist care provided by clinics under contract by region Stockholm that charge the same regulated out-of-pockets fees as publicly provided care. We did not include fully private specialist services where patients pay the full cost, as these fall outside the scope of public expenditure and is very uncommon in Sweden (0.6% of total care expenditures). Though we agree that examining private service use across income groups would be valuable, the extent of privately (out-of-pocket) paid health and social care is very small in Sweden, and our expenditure estimates only reflect services financed through the public systems.

Reviewer 1, comment: In addition, providing a list of the types of services included under health and social care expenditure would aid the interpretation of the results.
Response: The measure of healthcare expenditure included all publicly funded care services provided by Region Stockholm. Similarly, social care services included all publicly funded elderly care services organized and provided by the municipalities within Stockholm County. Home help services include both domestic (laundry, cleaning, food shopping etc.) and personal care (bathing, toileting, eating and maneuvering from bed).

Reviewer 1, comment: The authors should also clarify the rationale for selecting 2019 and focusing on a single year. If this choice was influenced by substantial policy changes around the period, this needs to be stated.
Response: Care expenditure was restricted to 2019 to not go into the COVID-19 pandemic years, which might affect care expenditure due to changes in care seeking patterns, due to public health recommendations introduced.
Action: The following was added to the Methods section
The total population register "Registret över totalbefolkning" (RTB) was used to define the study population which included (N=371,583) adults aged ≥65 years registered as living in Region Stockholm on January 1st, 2019. The year was selected to eliminate the impact from the COVID-19 pandemic.

Reviewer 1, comment: While the authors acknowledge the limitation of focusing on a single region, the interpretation of findings might be enhanced by considering rural-urban differences among older individuals (if feasible).
Response: Thank you for this valuable suggestion. Unfortunately, it was not feasible to examine rural–urban differences within the scope of this study due to data limitations, as we only have data for one region which does not allow for a consistent or meaningful classification of rural versus urban. We now explicitly acknowledge this as a limitation and note it as a potential avenue for future research.
Action: Added to the Discussion section.
The findings generalisability requires additional consideration, as care organisation is decentralized in Sweden, where regions and municipalities are highly autonomous in the financing and organising of health and social care services to their local populations (18). Additionally, rural–urban differences could not be examined meaningfully in this study, which is important as there are differences in care resources available (37) .

Reviewer 2, comment: Multimorbidity is defined as the cumulative diagnoses between 2016 and 2018, while care expenditure is limited to 2019, as stated in the abstract. Why was the cumulative expenditure from 2016 not considered? How can the expenditure from a single year accurately capture the overall burden, given that treatment costs vary significantly with disease progression?
Response: Thank you for this helpful comment. We decided when designing this study to focus on measuring the outcome care expenditure in 2019 to ensure that the exposure (multimorbidity measured based on diagnoses attached to care episodes between 2016-1=2018) clearly preceded the outcome, to limit reverse causation bias. As using cumulative expenditure from 201\6 onwards would have overlapped with the multimorbidity measurement window and risked capturing costs that occurred before or during the exposure period, which would blur the temporal ordering between the exposure and outcome. Our intention was to measure multimorbidity at baseline and influences on

subsequent care expenditure, and therefore, the measurement of care expenditure was restricted to the following year following the exposure window.
Action: The following was added to the Methods section
The total population register “Registret över totalbefolkning” (RTB) was used to define the study population which included (N=371,583) adults aged ≥65 years registered as living in Region Stockholm on January 1st, 2019. The year was selected to eliminate the impact from the COVID-19 pandemic.

Reviewer 2, comment: The measurement of multimorbidity should go beyond simply counting the number of diseases and consider the types of diseases and their interactions. For instance, a random combination like skin rash and hypertension differs significantly from a clinically relevant combination such as hypertension and coronary disease, which has greater impacts on health outcomes and costs. The lack of analysis on specific combinations limits the study's conclusions. The sharp rise in care expenditure for patients with 4+ comorbidities may be driven by cardiovascular, metabolic, or respiratory conditions, particularly in older populations. Identifying and analyzing the most common multimorbidity combinations, their association with care expenditure, and comparing these to average costs would provide deeper insights and support targeted prevention and management strategies.
Response: Thank you for this thoughtful and important comment. We agree that multimorbidity is a complex construct and that the inclusion of disease combination would be of greater clinical relevance. Moreover, using the number of conditions rather than analysing specific combinations does not capture the heterogeneity in disease patterns or their differential effects on health status and care expenditure. And you are correct to highlight that certain combinations of conditions (i.e., cardiovascular, metabolic, and respiratory clusters) are likely drivers of the higher care expenditure observed among those with four or more chronic conditions, particularly in older populations. While the current analysis focuses on a standardized multimorbidity count to maintain methodological consistency and comparability, we recognize that exploring disease clusters, clinically relevant combinations of chronic conditions, and their association with expenditure represents an important next step.
We have highlighted this in the discussion as an area for future research, and we appreciate your suggestion, which reinforces the value of moving beyond simple counts toward more nuanced multimorbidity phenotyping.
Action: The following has been added to the discussion:
As multimorbidity was measured using healthcare utilisation data in the years prior, which limits our ability to assess incidence of multimorbidity. Specifically, individuals transitioning from zero or one chronic condition into multimorbidity, and the increased expenditure associated with this transition were not captured (5). Multimorbidity is frequently defined as the co-occurrence of two or more chronic conditions in an individual. However, this cutoff based on the quantity of diagnoses does not reflect the severity of each individual chronic condition (1, 26). A systematic review found that some multimorbidity combinations are associated with disproportionately high costs, driven by frequent hospitalisations and extensive care needs. Including respiratory and mental health conditions, diabetes with cardiovascular disease, and cancer with mental health conditions (16). Future research should explore disease combinations linked to higher health and social care expenditure, to identify high-need patient groups and inform targeted, cost-effective service planning.

Reviewer 2, comment: "males have higher healthcare expenditures while females incur higher social care expenditures " and "the relative changes of care expenditure over time across different socio-economic groups" suggest that these patterns may be influenced by broader social and economic

dynamics (healthcare access inequities, diagnostic bias or societal stereotypes, etc.) . These results should be thoroughly discussed to uncover the underlying drivers, assess their implications for policy and practice in healthcare and social care systems.
Response: This study observed different expenditure patterns between men and women and across socioeconomic groups, likely reflecting the broader social and economic dynamics within the health-and-social care systems.
Action: The following has been added to the discussion:
Multimorbidity was more prevalent among females at earlier ages compared to males, moreover, there were sex differences in service specific expenditure, where females incurred higher social care expenditure whereas males incurred slightly higher healthcare expenditure. These differences in expenditure might be attributed to marked differences in disease progression, care-seeking behaviours, availability of informal support, and life expectancy as well as broader structural and social factors within care systems.

Reviewer 2, comment: While the research highlights dementia as a major driver of care home placement expenditure, this conclusion may be biased due to the lack of investigation into the disease types within multimorbidity. Dementia is undoubtedly a critical condition contributing to higher expenditures, other conditions or combinations of diseases may also significantly drive care home placement costs. Additionally, it would be valuable to explore multimorbidity patterns within dementia patients compared to those without to better understand the interplay between dementia and other comorbidities. Why was dementia singled out in this analysis?
Response: Thank you for this thoughtful comment. Dementia was highlighted because we observed that care home expenditure was higher among non-multimorbid individuals and because previous research has consistently shown dementia diagnosis to be the strongest predictor of social care utilization and care home admissions, due to its substantial impact on cognitive decline, functional dependency, and need for continuous supervision. So, while our study did not examine specific disease types or multimorbidity clusters, we acknowledge that other conditions-and-combinations of conditions may also significantly contribute to care home admissions and subsequent care expenditure. The singling out of dementia was not intended to imply exclusivity, but rather to contextualise our findings within a well-established driver of social care expenditure. We agree that comparing multimorbidity patterns among individuals with and without dementia would offer valuable additional insight, and this a direction we will consider including in future research.
Action: The sentence below has been added to the Methods section.
To assess the socioeconomic and demographic differences in multimorbidity and care expenditure, multimorbidity was defined as the presence of two or more chronic conditions, without distinguishing between specific disease combinations or patterns.
Action: The sentence below has been added to the Discussion section.
Future research should explore disease combinations linked to higher health and social care expenditure, to identify high-need patient groups and inform targeted, cost-effective service planning.

Reviewer 2, comment: "specialist outpatient care" is not clearly defined or not with listed examples;
Response: This term should be "Outpatient specialist care" refers to medical consultations, diagnostic tests and treatments provided by specialist medical doctors, provided in clinics or hospitals, allowing the patient to leave the same day, these services include for example cardiology, potamology, and minor surgeries.
Action: A description of outpatient specialist care has been added in the Methods section.

Reviewer 2, comment: I would recommend the authors thoroughly review the manuscript for typos,

grammatical errors, and inconsistencies in symbols or formatting. For example, in Introduction line 12, "with multimorbidity(11).." has duplicated comma.

Response: We thank the reviewer for noting this. The manuscript has been carefully revised to correct typos and grammatical errors, as well as consistent formatting throughout. The duplicated punctuation in the Introduction and similar minor issues have been corrected.

Reviewer 2, comment: In the Introduction, the burden of multimorbidity is repeatedly described as being associated with higher healthcare utilization and negative impacts on health and social care. It would be beneficial to present this information more concisely to avoid redundancy and enhance the clarity of the narrative.

Response: We thank the reviewer for this helpful suggestion. The Introduction has been revised to reduce redundancy by streamlining descriptions of the burden of multimorbidity and its implications for health and social care utilization, thereby improving clarity and narrative flow.

Reviewer 2, comment: Any historical diagnoses before 2016 could be possible. How to deal with this scenario?

Response: Thank you for this important point. Multimorbidity was measured using the validated register-based approach developed by Calderón-Larrañaga et al., which captures chronic conditions regardless of year of onset, reflecting their long-term and persistent nature. Diagnoses recorded during healthcare contacts between 2016–2018 could therefore include conditions that originated prior to 2016, consistent with the intended use of this measure. This approach reduces under-ascertainment of longstanding chronic diseases and aligns with the conceptualisation of multimorbidity as a cumulative condition burden.

Action: We have clarified this in the Methods section

Multimorbidity was measured using ICD-10 codes recorded in outpatient and inpatient care between 2016 and 2018. Because multimorbidity reflects a long-standing and cumulative disease burden, chronic conditions diagnosed prior to 2016 could still be captured through healthcare contacts during the observation period.

Reviewer 2, comment: Dose the Region Stockholm Healthcare administrative database (VAL) fully captures all inpatient care, outpatient care, and private practice records, or only partially. If the database does not include all records, this limitation should be explicitly stated.

Response: The Region Stockholm Healthcare administrative database (VAL) is a collection of registers that include information on all health and medical care that is reimbursed by region Stockholm. This database includes records of all inpatient care, outpatient care and care by licensed private practices. In Sweden, it is the regional databases that provide information for national statistics and the nationwide patient registers. The use of VAL in this study is a strength as it allows for the estimating of public expenditure on a wide range of healthcare services, including primary care and home-healthcare services which are not included in the national patient register.

Reviewer 2, comment: Regarding the 297,16 patients without expenditure data, please clarify whether this is due to data issues, such as incomplete linkage or missing records, or other factors. When linking multiple databases, it is critical to perform rigorous data cleaning and quality assurance to ensure the integrity and reliability of the data used for analysis. Explicitly documenting these steps will help ensure high-quality data and robust conclusions.

Response: The n=29,716 individuals identified as having no expenditure data did not reflect missing records or incomplete data linkage. Rather, these individuals incurred zero recorded health or social

care expenditure during 2019. This was identified because the linked administrative datasets used in this study provided complete population-level coverage for publicly funded health and social care services within the study context. Linkage between registers was performed using encrypted serial numbers as identifiers, followed by systematic data cleaning and validation procedures, including checks of duplicate records and inconsistent identifiers. As a result, the absence of expenditure for these individuals reflects true non-utilisation of recorded services during the study period rather than data quality issues.

Reviewer 2, comment: "Model 0" is not defined in Method.
Response: Model 0 examined the association between multimorbidity and total health and social care expenditure without adjusting for any other factors.
Action: Model 0 has been defined in the Methods section under the sub-heading Analysis.

Reviewer 2, comment: The 2nd and 3rd phragraph of Results have mainly state as "higher" or "lower", but no explict numbers ever listed.
Action: This text has been edited.
Figure 1 provides an overview of the average expenditure on health and social care services across ages. Average health and social care expenditure increased with older age, and from ≥80 years onwards social care expenditure exceeded healthcare expenditure. Among adults ≥80 years, a larger proportion of the healthcare expenditure consisted of home healthcare, as compared to those aged 65-79 years. From 90 years, the average expenditure on specialist care and emergency care decreased.

[Insert Figure 1.]

Figure 2 descriptively shows care expenditure by care service according to multimorbidity status. Across ages, individuals with multimorbidity (represented by the blue line) had greater average inpatient, outpatient, and home healthcare expenditure compared to those without multimorbidity (black dashed line), with expenditure increasing with age. Differences in emergency care expenditure were most pronounced at 65 years. From 80 years onwards, care home expenditure was higher among non-multimorbid individuals. This is explored further in Figure S5, where care home placement expenditure was greater among those with dementia. Home-help expenditure was greater among those with multimorbidity. Figure S6 presents total care expenditure (i.e., health and social care) by income group, with marked age-related increases in social care expenditure and modest increases in healthcare expenditure. Lower income groups had higher care expenditures at younger ages compared to higher income groups. Moreover, larger differences across income groups were observed for social care expenditure.

VERSION 2 - REVIEW

**Reviewer**	**1**
**Name**	**Hamashima, Yuri**
**Affiliation**	**University of Bristol, NIHR ARC West, Population Health Sciences**
**Date**	**18-May-2026**
**COI**	

Thank you for the opportunity to review the revised manuscript. I can see that the authors addressed the questions and suggestions raised and revised the manuscript accordingly. I have no further comments and would recommend the paper for acceptance.
