# Supplementary material for: Socioeconomic differentials in multimorbidity prevalence, health and social care expenditure among persons 65 years and older in region Stockholm, Sweden: a population-based study
Source: BMJ Open. 2026 Aug 14;16(8):e106179. doi: 10.1136/bmjopen-2025-106179 (PMC13479488; doi:10.1136/bmjopen-2025-106179)
Supplement: Supplementary file 2 [file bmjopen-16-8-s001.docx]

## **SUPPLEMENTARY MATERIAL**

**Figure S1:** Assessment of the plots of the residuals assessed to determine the best approach to modelling total care expenditure

**Table S1:** Model selection based on comparison of fit statistics to select the most appropriate generalized linear model to assess the effect of multimorbidity on total care expenditure.

|  | Total care expenditure | | | | Natural log of total care expenditure | | | |
| --- | --- | --- | --- | --- | --- | --- | --- | --- |
|  | Log Likelihood | df | AIC | BIC | Log Likelihood | df | AIC | BIC |
| Model 0 |  |  |  |  |  |  |  |  |
| Gamma distribution, log link | -4580792 | 1 | 9161587 | 9161598 | -1112622 | 1 | 2225247 | 2225258 |
| Model 2 |  |  |  |  |  |  |  |  |
| Gamma distribution, log link | -4138528 | 9 | 8277073 | 8277170 | -1108876 | 9 | 2217771 | 2217868 |
| Guassian distribution, log link | -4663422 | 9 | 9326862 | 9326958 | -690026.4 | 9 | 1380071 | 1380167 |
| Gamma distribution, power link | -4140406 | 9 | 8280829 | 8280926 | -1108878 | 9 | 2217773 | 2217870 |
| Poisson distribution, log link | -3.54E+10 | 9 | 7.08E+10 | 7.08E+10 | -757651.2 | 9 | 1515320 | 1515417 |

*df = degrees of freedom, AIC=Akaike Information Criteria, BIC= Bayesian Information Criteria. *Model 0= multimorbidity, *Model 2= multimorbidity+ income group + age in years + sex + country of birth. (N=341,867)

After comparing the plots of the residuals in figure S1, the model fit was assessed for the outcome of total care expenditure and the natural log of total care expenditure. The final model selected was a GLM model with a gamma distribution and log link and the outcome was transformed as this had the best loglikelihood (-1108876) and corresponding AIC.

**Table S2:** Characteristics of individuals in the study population with zero care expenditure in 2019

|  | Population |
| --- | --- |
|  | n=29,716 |
| *Sex* |  |
| Male | 52.5% |
| Female | 47.5% |
| *Age groups* |  |
| 65-69 yrs | 45.3% |
| 70-74 yrs | 30.7% |
| 75-79 yrs | 13.9% |
| 80-84 yrs | 5.9% |
| 85-89 yrs | 2.5% |
| ≥90yrs | 1.7% |
| *Income groups* |  |
| (lowest) group 1 | 29.7% |
| group 2 | 20.6% |
| group 3 | 13.6% |
| group 4 | 13.7% |
| (highest) group 5 | 19.9% |
| missing | 2.4% |
| *Country of birth* |  |
| Other | 32.4% |
| Sweden | 67.6% |
| *Multimorbidity* |  |
| None | 73.3% |
| 1 condition | 15.2% |
| 2 conditions | 6.6% |
| 3 conditions | 2.6% |
| ≥4 conditions | 2.3% |

**Figure S2:** Prevalence of multi-morbidity by age in years, stratified by sex, among adults ≥65 years old in Region Stockholm 2019. Blue=male, red=female.

**Figure S3:** Prevalence of multimorbidity by income and age in adults ≥65 years old in Region Stockholm 2019. Income group 1 (blue)=the lowest income group; income group 5 (red)=the highest income group.

**Figure S4:** Prevalence of multi-morbidity across income groups stratified by sex (A=female, B=male) and age in years among adults aged ≥65 years living in Region Stockholm in 2019. Blue=group 1 (the lowest income group), yellow=group 2, purple=group 3, green=group 4, red=group 5 (the highest income group).

 **Figure S5:** Average expenditure for all care (total care costs), healthcare and social care, by income group among adults ≥65 years in region Stockholm in 2019. Income group 1 (blue)=the lowest income group; income group 5 (red)=the highest income group.

**Figure S6:** Average expenditure on care home placement across ages stratified by people without a dementia diagnosis represented by the blue line and with a dementia diagnosis represented by black dashed line.
